# Dealing with professional misconduct by colleagues in home care: a nationwide survey among nursing staff

**DOI:** 10.1186/s12912-016-0182-2

**Published:** 2016-10-12

**Authors:** Erica E. M. Maurits, Anke J. E. de Veer, Peter P. Groenewegen, Anneke L. Francke

**Affiliations:** 1 Netherlands Institute for Health Services Research (NIVEL), P.O. Box 1568, 3500 BN Utrecht, The Netherlands; 2 Department of Sociology and Department of Human Geography, Utrecht University, Utrecht, The Netherlands; 3 Department of Public and Occupational Health, EMGO Institute for Health and Care Research (EMGO+), VU University Medical Center, Amsterdam, The Netherlands

**Keywords:** Professional misconduct, Impairment, Incompetence, Home care, Nursing

## Abstract

**Background:**

Professional misconduct in healthcare, a (generally) lasting situation in which patients are at risk or actually harmed, can jeopardise the health and well-being of patients and the quality of teamwork. Two types of professional misconduct can be distinguished: misconduct associated with incompetence and that associated with impairment. This study aimed to (1) quantify home-care nursing staff’s experiences with actual or possible professional misconduct; (2) provide insight into the difficulty home-care nursing staff experience in reporting suspicions of professional misconduct within the organisation and whether this is related to the individual characteristics of nursing staff; and (3) show which aspects of professional practice home-care nursing staff consider important in preventing professional misconduct.

**Methods:**

A questionnaire survey was held among registered nurses and certified nursing assistants employed in Dutch home-care organisations in 2014. The 259 respondents (60 % response rate; mean age of 51; 95 % female) were members of the Dutch Nursing Staff Panel, a nationwide group of nursing staff members in various healthcare settings.

**Results:**

Forty-two percent of the nursing staff in home care noticed or suspected professional misconduct by another healthcare worker during the previous year, predominantly a nursing colleague. Twenty to 52 % of the nursing staff experience difficulty in reporting suspicions of different forms of incompetence or impairment. This is related to educational level (in the case of incompetence), and managerial tasks (both in the case of incompetence and of impairment). Nursing staff consider a positive team climate (75 %), discussing incidents (67 %) and good communication between healthcare workers (57 %) most important in preventing professional misconduct among nursing staff.

**Conclusions:**

Suspicions of professional misconduct by colleagues occur quite frequently among nursing staff. However, many nursing staff members experience difficulty in reporting suspicions of professional misconduct, especially in the case of suspected impairment. Home-care employers and professional associations should eliminate the barriers that nursing staff may encounter when they attempt to raise an issue. Furthermore, advocating a positive team climate within nursing teams, encouraging nursing staff to discuss incidents and facilitating this, and promoting good communication between healthcare workers may be appropriate strategies that help reduce professional misconduct by nursing staff.

## Background

Professional misconduct among home-care nursing staff can jeopardise the health and well-being of patients and the quality of nursing staff’s teamwork. Therefore, it is important to address and prevent professional misconduct in home care. Professional misconduct in health care is defined by the Dutch Health Care Inspectorate as a (generally) lasting situation in which patients are at risk or actually harmed because a healthcare worker lacks competencies or provides irresponsible care and seems unable or unwilling to change this situation [1]. Two types of professional misconduct can be distinguished, viz. misconduct associated with incompetence and misconduct associated with impairment [2, 3]. In the case of incompetence, a healthcare worker’s functioning is harmed as a result of a deficiency in knowledge or skills, including communication and collaboration problems [3]. In the case of impairment, a healthcare worker’s cognitive, interpersonal or psychomotor abilities are seriously impaired due to individual conditions that interact with the environment (e.g. substance abuse, aggressive behaviour, mental illness or physical disability) [4, 5]. Impairment is frequently accompanied by incompetence [3].

In home care, nursing staff play an important role in caring for the chronically ill and the elderly. Home-care patients in general depend heavily on the care they receive and may therefore be reluctant to report professional misconduct. Signs of incompetence or impairment that are perceived and acted upon by nursing colleagues may therefore play an important role in reducing professional misconduct. However, since staff in home-care nursing generally deliver care individually in the homes of their clients, it may be more difficult for nursing staff to identify possible professional misconduct by colleagues than in other healthcare sectors.

To our knowledge, there have been no studies that quantify home-care nursing staff’s experiences with actual or possible professional misconduct as a (generally) lasting situation. Previous research has concentrated on *instances* of inadequate care or disruptive behaviour and predominantly focussed on in-patient care. These studies revealed that nurses quite frequently notice such *instances*. Moore and McAuliffe [6] showed that 88 % of the nurses in acute-care hospitals had observed an incident of poor care in the past 6 months. Rosenstein and O’Daniel [7] found that 72 % of the hospital nurses had at some point witnessed disruptive behaviour from another nurse at their hospital. Lastly, Malmedal, et al. [8] showed that 91 % of the nursing staff in nursing homes had at some point observed an act of inadequate care committed by a colleague. The first objective of our study was to quantify home-care nursing staff’s experiences with actual or possible professional misconduct.

It is important that home-care nursing staff report observations of professional misconduct by nursing colleagues, either formally or informally, in order to tackle professional misconduct and prevent any further harm to patients. According to the International Council of Nurses’ code of ethics, nurses have to take appropriate action to safeguard patients when their health is endangered by a colleague [9]. Nonetheless, nurses do not always report acts of inadequate care committed by their colleagues [6, 10]. Seeing a colleague not functioning properly gives rise to high levels of moral distress among nursing staff [11]. Nursing staff’s reluctance to report incidents may also apply to suspicions of professional misconduct. Frequently named factors that deter nurses from reporting incidents of poor care or raising concerns about patient safety are the belief that their concerns will be ignored, not wanting to cause trouble, and fear of retribution or repercussions [6, 10, 12]. However, these factors may be most relevant for nurses in hospitals, where the division of power between physicians and nursing staff may affect nurses’ reporting behaviour.

It is likely that home-care nursing staff experience more difficulty in reporting suspicions of impairment than in reporting supposed incompetence. Signs of impairment may be less clear and more of a taboo subject than indications of incompetence, since impairment concerns personal problems and incompetence involves professional functioning that has deteriorated due to a deficiency in knowledge or skills. In addition, it is plausible that communication about professional knowledge and skills is common within teams, while personal problems are discussed less often.

Furthermore, it can be expected that there are individual differences in the reluctance to report suspicions of incompetence or impairment and that these differences partly depend on professionalism, position within the organisation and prior experience with professional misconduct. First, it is plausible that registered nurses with a bachelor’s degree experience less difficulty in reporting suspicions of professional misconduct by colleagues than nursing staff with a lower level of educational attainment. Registered nurses with a bachelor’s degree can be characterised as having a higher degree of professionalism, as they perceive more autonomy in their work than nursing staff in home care with a lower level of education [13]. Autonomy is viewed as a core characteristic of professionalism in the literature on professions (e.g. [14, 15]). Self-regulation by a professional group is important to prevent autonomy from resulting in professional misconduct. Professions can be viewed as moral communities whose norms, values and definitions of appropriate professional conduct guide the individual professionals in their work ([14], p.25, p.100). This moral dimension helps to sustain society’s trust in the professions [16]. Although national codes of ethics may exist for nursing staff with both lower and higher levels of education, as is the case in the Netherlands, it stands to reason that registered nurses with a bachelor’s degree are more accustomed to exerting social control over the behaviour of colleagues. Also, as suggested by Malmedal, et al. [17], more highly educated nursing staff may have had more training in critically reflecting upon practice and have acquired more knowledge about ethics and moral practice.

Secondly, it can be expected that more experienced nursing staff have less difficulty in reporting incompetence or impairment. These nursing staff may have a better sense of the limits of permissible behaviour in nursing. However, an opposite effect could also be postulated. Less experienced nursing staff may have more optimistic views on the likely outcome of reporting suspicions of professional misconduct by nursing colleagues. Nursing staff members with more work experience may have experienced or heard of disappointing results from reporting professional misconduct. Thirdly, it can be assumed that home-care nursing staff with managerial tasks experience less difficulty in reporting suspicions of professional misconduct by colleagues since they are more accustomed to judging the performance of colleagues and are better connected to senior management. Finally, it is likely that nursing staff who have actually experienced or suspected professional misconduct by a nursing colleague report more difficulty in voicing suspicions of impairment or incompetence than nursing staff lacking this experience. Nursing staff without actual experience with actual or suspected misconduct may underestimate the moral distress that is triggered by these situations.

Insight into these differences can help home-care employers and professional associations in tailoring their policies to tackle professional misconduct by nursing staff. Hence, the second objective of this study was to provide insight into the difficulty that home-care nursing staff experience in reporting suspicions of professional misconduct by nursing colleagues, and the individual characteristics that are related to this difficulty.

In addition to dealing appropriately with nursing staff members who demonstrate incompetence or impairment, it is important to prevent professional misconduct wherever possible. It would thus be of interest to know which aspects of professional practice home-care nursing staff consider important in preventing professional misconduct. With this knowledge, home-care employers and professional associations can strengthen their policies for curbing professional misconduct. However, no research has been found that surveyed nursing staff’s views on practice factors that help to prevent professional misconduct. The third objective of the current study was to explore different aspects of professional practice that nursing staff consider important in preventing professional misconduct, and possible associations between this and the educational level of nursing staff or their prior experience with misconduct.

The main questions addressed in this study are:

1. To what extent do home-care nursing staff have experience with nursing colleagues who were or suspected to be incompetent or impaired?
2. To what extend do home-care nursing staff experience difficulty in reporting suspicions of professional misconduct by nursing colleagues? Do nursing staff experience less difficulty in reporting suspicions of incompetence than in reporting suspicions of impairment?
3. Is difficulty in reporting suspicions of professional misconduct related to educational level, work experience, managerial tasks and prior experience with professional misconduct?
4. Which aspects of professional practice do home-care nursing staff consider important in preventing professional misconduct by nursing colleagues?
5. Are educational level and prior experience with professional misconduct related to the aspects of professional practice that nursing staff consider important?

## Methods

### Design and setting

This study used a quantitative, exploratory design. Data were collected by a questionnaire survey among home-care registered nurses and certified nursing assistants in the Netherlands in May and June 2014. Dutch home-care services include support in daily living activities (i.e. personal care), technical nursing care and psychosocial care, all of which are delivered mainly by registered nurses and certified nursing assistants. This home care can be episodic, e.g. after a hospital stay, but it is more often longer lasting [18]. The education of Dutch certified nursing assistants consists of 3 years of vocational training after secondary education. This is different from the situation in most other countries, where nursing assistants often have vocational training of less than 1 year. Dutch registered nurses are educated to two different levels. Nurses educated to associate degree level have had 3 to 3.5 years of professional training (equivalent to a UK foundation qualification) and nurses educated to bachelor’s degree level have had at least 4 years of professional training [18, 19].

In the Netherlands, a national code of ethics applies to both certified nursing assistants and registered nurses [20]. This code requires nursing staff to point out acts of poor care or harmful behaviour to the healthcare worker concerned. If a conversation with the healthcare worker does not have the desired effect, nursing staff should inform the appropriate person or authority (e.g. the supervisor, manager, complaints committee, medical disciplinary law judge or Dutch Health Care Inspectorate) while taking due care.

### Sample

A total of 259 Dutch nursing staff working in home care completed the questionnaire (response rate of 60 %). All respondents were members of a pre-existent research sample, the Nursing Staff Panel, consisting of a nationwide group of nursing staff members in various healthcare settings who deliver direct patient care and are willing to fill in questionnaires about current topics in health care.

Members of the Nursing Staff Panel are recruited via a random sample of the population of Dutch healthcare employees provided by the Dutch Employee Insurance Agency. This agency is responsible for social security payments and registers all employees in the Dutch healthcare sector. Healthcare employees in this random sample were asked to participate in healthcare research for various purposes. Nursing staff delivering direct patient care in the largest healthcare sectors in the Netherlands (i.e. hospitals, mental health care, care for disabled people, home care, nursing homes and homes for the elderly) who agreed to this request were invited to become members of the Nursing Staff Panel. This procedure promotes a diverse composition of the panel with respect to age, gender, region and employer. Participation in the Nursing Staff Panel is voluntary and takes place on an anonymous basis.

### Data collection

The questionnaire was administered in Dutch. Respondents could complete the questionnaire online or on paper. To increase the response rate, up to two reminders were sent at fortnightly intervals to panel members who had not yet responded.

The questionnaire contained both self-developed questions and questions from an existing questionnaire on dealing with colleagues demonstrating impairment or incompetence in health care by Weenink, et al. [3], adjusted to suit nursing staff. The questionnaire was assessed by five experts in nursing research and ethics in health care and amended to take account of their comments.

#### Experience with colleagues who demonstrated actual or possible incompetence or impairment

The questionnaire asked nursing staff whether they had noticed or suspected professional misconduct by another healthcare worker in the preceding 12 months. To ensure respondents would interpret the concept of professional misconduct appropriately, they were shown the Health Care Inspectorate’s definition of professional misconduct as described above in the Background section [1]. A comment was added that professional misconduct can also involve substance abuse or transgressive behaviour. The question was derived from the questionnaire by Weenink, et al. [3]. Possible responses were ‘yes’ and ‘no’. If the answer was ‘yes’, respondents were asked to specify the profession of this healthcare worker. Their responses were categorised into 1) nursing colleague, 2) physician, 3) other healthcare worker. If respondents had noticed or suspected professional misconduct more than once, they were asked to report the most recent case. Respondents who had noticed or suspected professional misconduct were also asked to specify the type of professional misconduct. They were shown four categories of incompetence and seven categories of impairment (see Table 3). The categories of incompetence and impairment were derived from the questionnaire by Weenink, et al. [3], although two additional categories were included – ‘Dealing carelessly with patient’s personal belongings’ and ‘Fraud’ – on the advice of the aforementioned experts. Multiple responses were possible. Respondents could also mention another type of professional misconduct.

#### Difficulty in reporting suspicions of professional misconduct by colleagues

For each of the 11 categories of professional misconduct (see Table 3), respondents were requested to picture that they suspect a nursing colleague of this particular type of incompetence or impairment. A ‘nursing colleague’ was defined as a nurse or nursing assistant in the same home-care team. Respondents were subsequently asked to indicate whether they would find it easy or difficult to raise the matter of this suspicion within their organisation. The responses were on a five-point Likert scale and coded as 1 = ‘very easy’, 2 = ‘easy’, 3 = ‘neither easy nor difficult’, 4 = ‘difficult’ and 5 = ‘very difficult’. A mean score was calculated for both the four incompetence categories and the seven impairment categories, resulting in two scale scores, with a possible range of 1–5.

#### Important aspects of professional practice in preventing professional misconduct

Respondents were shown 16 aspects of professional practice and asked to indicate which of these aspects they consider as most important in preventing professional misconduct by nursing colleagues (see Table 1). Respondents were requested to select up to five aspects. They could also add an additional aspect they considered important to the list of aspects.

**Table 1:** Aspects of professional practice that could be important in preventing professional misconduct, as addressed in the questionnaire

Aspects of professional practice
Support and guidance by nurse managers
Transparent communication patterns
Regular performance appraisal interviews
Individual work support or supervision
Supplementary training
Personal development plan
Peer review
Discussing incidents
Using professional profiles/competence profiles/professional codes of ethics
Good communication between healthcare workers
Positive team climate/culture of openness
Flexibility in working hours
Ability to work reduced hours over a period of time if necessary
Sufficient staff
Suitably qualified staff

#### Respondents’ characteristics

The respondent characteristics addressed in the survey questionnaire are age, sex, educational level, managerial tasks, work experience in health care, and working hours per week. Educational level was specified as the highest level of nursing education completed (certified nursing assistant, registered nurse with an associate-level degree or registered nurse with a bachelor’s degree). Work experience was defined as the number of years practicing as a registered nurse or certified nursing assistant. Respondents were classified into four age groups (see Table 2). Both years of work experience and number of working hours per week were categorised into four groups.

**Table 2:** Summary of respondent characteristics (*n* = 259)

	% or mean (*S.D.*)	Missing data (%)
Age (years)	50.79 (9.30)	0 %
< 40	13.1 %	
40–49	22.8 %	
50–59	48.7 %	
> 59	15.4 %	
Sex		0 %
Male	4.3 %	
Female	95.8 %	
Educational level		0.8 %
Certified nursing assistant	51.0 %	
Registered nurse, associate-level degree	25.7 %	
Registered nurse, bachelor’s degree	23.4 %	
Managerial tasks^a^		1.2 %
No	86.3 %	
Yes	13.7 %	
Work experience (years)	23.00 (10.75)	6.6 %
< 10	13.6 %	
10–19	21.1 %	
20–29	31.0 %	
> 29	34.3 %	
Working hours per week	22.7 (7.50)	6.6 %
< 16	14.9 %	
16–23	33.5 %	
24–31	36.4 %	
> 31	15.3 %	

^a^ Managerial tasks are performed mainly by registered nurses. Only 0.8 % of the certified nursing assistants have managerial tasks, while 12 % of the registered nurses with an associate degree and 40 % of the nurses with a bachelor’s degree have managerial tasks

### Data analysis

The data were analysed using Stata 13.1. The level of statistical significance was set at *p* < 0.05. Descriptive statistics were calculated for experience with actual or suspected professional misconduct by colleagues, difficulty in reporting suspicions of professional misconduct by colleagues and aspects of professional practice that nursing staff consider important in preventing professional misconduct. Independent t-tests (for dichotomous independent variables) and analyses of variance (for independent variables with more than two categories) were conducted to explore bivariate relationships between individual characteristics and difficulty in reporting suspicions of professional misconduct. Additional regression analysis was performed to check for possible interaction and interdependence between individual characteristics with *p* < 0.05 in the bivariate analyses. A dependent t-test was performed to compare the difficulty in reporting suspicions of incompetence and of suspicions of impairment. Bivariate relationships between educational level and experience with professional misconduct on the one hand and aspects of professional practice that nursing staff members consider important in preventing professional misconduct on the other hand, were examined using Pearson’s chi-square tests.

## Results

### Respondents’ characteristics

The individual characteristics of the respondents are shown in Table 2. Most were female (95 %). The respondents ranged in age from 23 to 66. Their average age of 51 (standard deviation or *S.D.* = 9.3) was higher than the average age of employees working in the home-care sector in the Netherlands, which was 44 in 2014 [21]. The majority of the respondents (51 %) were certified nursing assistants, 26 % had an associate-level degree in nursing and 23 % a bachelor’s degree. As the respective proportions in the Dutch home-care sector as a whole were 69 %, 17 % and 14 % in 2014 [22], certified nursing assistants were under-represented and registered nurses were over-represented in the study population. We have addressed the slightly distorted distribution of the sample by performing subgroup analyses. Respondents had 23 years of experience in nursing on average (*S.D.* = 10.8) and an average weekly working time of 23 h (*S.D.* = 7.5). Most respondents (86 %) delivered only direct patient care, while 14 % also had managerial tasks.

### Experience with actual or suspected misconduct by another healthcare worker

As shown in Table 3, 42 % (95 % confidence interval of 36–48 %) of the respondents reported that they had noticed or suspected professional misconduct by another healthcare worker in the past 12 months. No statistically significant differences in experience with misconduct were found between age groups and educational levels. In most cases of actual or suspected professional misconduct (85 %), the healthcare worker concerned was a nursing colleague. 11 % of the respondents who had experienced actual or suspected professional misconduct said that the healthcare worker concerned was a physician. Furthermore, most respondents (56 %) indicated that the professional misconduct entailed substandard care, followed by communication problems with colleagues (42 %) and collaboration problems with colleagues (38 %). The different types of impairment were mentioned far less often (10 % or less).

**Table 3:** Experience with a healthcare worker demonstrating actual or possible incompetence or impairment during the past 12 months

	%	*n*
Experience with a healthcare worker demonstrating actual or possible incompetence or impairment		
Total	41.7 %	259
Educational level^a^		257
Certified nursing assistant	39.7 %	
Registered nurse, associate-level degree	40.9 %	
Registered nurse, bachelor’s degree	45.0 %	
Age (years)^b^		259
< 40	47.1 %	
40–49	39.0 %	
50–59	42.9 %	
> 59	37.5 %	
Category of healthcare worker demonstrating incompetence or impairment		98
Nursing colleague	84.7 %	
Physician	11.2 %	
Other	4.1 %	
Category of incompetence or impairment (multiple responses possible)		106
Incompetence		
Substandard care	55.7 %	
Collaboration problems with colleagues	37.7 %	
Communication problems with colleagues	41.5 %	
Communication problems with patients	34.0 %	
Impairment		
Careless handling of patient’s belongings	3.8 %	
Fraud	3.8 %	
Substance abuse (e.g. drugs or alcohol)	1.9 %	
Aggressive behaviour (verbally or physically)	2.8 %	
(Sexually) inappropriate behaviour or remarks	0.9 %	
Physical impairment	10.4 %	
Mental illness	8.5 %	
Other	9.4 %	

^a^No statistically significant differences in experience with misconduct were found between educational levels (Chi^2^ (2) = 0.48, *p* = 0.786)

^b^No statistically significant differences in experience with misconduct were found between age groups (Chi^2^ (3) = 0.94, *p* = 0.816)

### Difficulty in reporting suspicions of professional misconduct by colleagues

As presented in Table 4, 20 to 35 % of the nursing staff regarded reporting suspicions of different types of incompetence as difficult or very difficult. Reporting suspicions of different types of impairment was considered difficult or very difficult by 25 to 52 % of the nursing staff. Nursing staff experience most difficulty in reporting suspicions of substance abuse and fraud (both categories of impairment). The dependent t-test showed that nursing staff experience more difficulty in reporting suspicions of impairment (mean score of 3.18) than in reporting suspicions of incompetence (mean score of 2.95).

**Table 4:** Difficulty in raising the matter of suspicions of professional misconduct by a colleague (*n* = 236 to 238)

	Mean score (range 1–5)	Very easy	Easy	Neither easy nor difficult	Difficult	Very difficult
Incompetence	2.95^a^					
Substandard care	3.02	2.9 %	31.5 %	30.3 %	31.5 %	3.8 %
Collaboration problems with colleagues	3.01	1.3 %	29.8 %	38.7 %	27.3 %	2.9 %
Communication problems with colleagues	3.01	1.7 %	28.6 %	40.3 %	25.6 %	3.8 %
Communication problems with patients	2.77	3.4 %	38.4 %	38.0 %	18.6 %	1.7 %
Impairment	3.18^a^					
Careless handling of patient belongings	2.80	4.2 %	38.7 %	32.4 %	22.7 %	2.1 %
Fraud	3.36	3.0 %	22.5 %	24.6 %	36.0 %	14.0 %
Substance abuse (e.g. drugs or alcohol)	3.43	2.1 %	16.9 %	29.1 %	39.2 %	12.7 %
Aggressive behaviour (verbally or physically)	3.20	3.8 %	24.1 %	28.7 %	35.0 %	8.4 %
(Sexually) inappropriate behaviour or remarks	3.23	3.8 %	23.6 %	27.4 %	35.9 %	9.3 %
Physical impairment	2.95	2.1 %	34.2 %	35.0 %	24.1 %	4.6 %
Mental illness	3.30	1.3 %	21.5 %	32.1 %	36.7 %	8.4 %

^a^On average, nursing staff find reporting impairment more difficult than reporting incompetence, *t* (235) = -5.09, *p* = 0.000

An analysis of variance and a t-test showed that difficulty in reporting suspicions of incompetence is related to educational level and managerial tasks (Table 5). Certified nursing assistants and registered nurses with an associate-level degree experience more difficulty than registered nurses with a bachelor’s degree. Nursing staff without managerial tasks experience more difficulty in reporting suspicions of incompetence than nursing staff with managerial tasks. Additional regression analysis (not in table) revealed that when controlling for educational level, performing managerial tasks remains significantly related to difficulty in reporting suspicions of incompetence. Since managerial tasks occur rarely among certified nursing assistants (see footnote to Table 2), they were excluded from this analysis. Furthermore, when controlling for managerial tasks, we still found an association of -.22 between educational level (associate degree versus bachelor’s degree) and difficulty in reporting suspicions of incompetence, although this relationship was not statistically significant. The lack of statistical significance is likely due to reduced statistical power since there were relatively few nurses with managerial tasks (see Table 2). Supplementary regression analysis showed no interaction between educational level and managerial tasks. In summary, the additional regression analyses confirm that both educational level and managerial tasks are related to difficulty in reporting suspicions of incompetence. Furthermore, the effect of having managerial tasks is the same among nurses with an associate degree as among nurses with a bachelor’s degree.

**Table 5:** Bivariate relationships between individual characteristics and difficulty in raising the matter of suspicions of professional misconduct (*n* = 232 to 237)

	Incompetence (range 1–5)			Impairment (range 1–5)		
	Mean	*t* or *F*	*p*	Mean	*t* or *F*	*p*
Educational level		4.16	0.017*^a^		0.68	0.505
Certified nursing assistant (CNA)	3.00			3.20		
Registered nurse, associate-level degree (RN-a)	3.08			3.23		
Registered nurse, bachelor’s degree (RN-b)	2.71			3.07		
Work experience in years		2.53	0.058		1.96	0.120
< 10	2.73			3.15		
10–19	2.84			3.11		
20–29	3.11			3.37		
> 29	2.97			3.06		
Managerial tasks		3.82	0.000*		2.45	0.015*
No	3.03			3.24		
Yes	2.51			2.87		
Experience with professional misconduct		-1.08	0.283		-0.93	0.353
No	3.00			3.12		
Yes	2.89			3.22		
Age		0.98	0.404		2.11	0.100
< 40	2.95			3.40		
40–49	3.06			3.30		
50–59	2.97			3.13		
> 59	2.78			2.98		
Working hours per week		1.84	0.140		1.84	0.141
< 16	3.11			3.32		
16–23	3.06			3.30		
24–31	2.84			3.11		
> 31	2.85			2.97		

^a^ A post-hoc Bonferroni test showed that CNA and RN-a staff experience more difficulty than RN-b staff (*p* < 0.05)

* Statistically significant

With regard to difficulty in reporting suspicions of impairment, only an association with managerial tasks was found (Table 5). Home-care nursing staff without managerial tasks experience more difficulty in reporting suspicions of impairment than nursing staff with managerial tasks.

### Important aspects of professional practice in preventing professional misconduct

As shown in Table 6, nursing staff considered a positive team climate (reported by 75 %), discussing incidents (reported by 67 %), and good communication between healthcare workers (reported by 57 %) as the most important aspects of professional practice in preventing professional misconduct by nursing colleagues. Furthermore, almost half of the nursing staff (49 %) believed support and guidance by nurse managers is important in order to prevent professional misconduct.

**Table 6:** Aspects of professional practice reported as important in preventing professional misconduct by at least 25 % of the nursing staff; differences depending on educational level and experience with professional misconduct (*n* = 235 to 236)

	Total	Educational level					Experience with professional misconduct			
		CNA	RN-a	RN-b	*Chi* ^*2*^	*p*	Yes	No	*Chi* ^*2*^	*p*
Positive team climate/culture of openness	75.4 %	77.5 %	74.1 %	71.9 %	0.70	0.704	72.9 %	77.1 %	0.55	0.459
Discussing incidents	67.4 %	68.3 %	69.0 %	64.9 %	0.27	0.875	66.7 %	67.9 %	0.04	0.848
Good communication between healthcare workers	57.2 %	58.3 %	60.3 %	50.9 %	1.22	0.542	54.2 %	59.3 %	0.61	0.435
Support and guidance by nurse managers	49.2 %	52.5 %	51.7 %	40.4 %	2.45	0.293	47.9 %	50.0 %	0.10	0.753
Transparent communication patterns	40.7 %	47.5 %	43.1 %	24.6 %	8.58	0.014*	42.7 %	39.3 %	0.28	0.599
Sufficiently qualified staff	40.7 %	35.8 %	43.1 %	49.1 %	2.99	0.225	45.8 %	37.1 %	1.78	0.182
Regular performance appraisal interviews	34.3 %	35.0 %	34.5 %	31.6 %	0.21	0.901	40.6 %	30.0 %	2.85	0.091
Individual work support or supervision	25.9 %	24.2 %	25.9 %	28.1 %	0.31	0.855	30.2 %	22.9 %	1.61	0.205

* Statistically significant

A chi-square test revealed that certified nursing assistants and registered nurses with an associate-level degree attach greater importance to transparent communication patterns than registered nurses with a bachelor’s degree. No further associations with educational level were found. Additionally, none of the professional practice aspects showed an association with prior experience with professional misconduct. In other words, nursing staff members with prior experience with actual or possible professional misconduct and staff without such experience attach the same importance to the different aspects of professional practice in preventing professional misconduct.

## Discussion

### Main findings

This study shows that, during the past year, 42 % of the nursing staff in home care noticed or suspected professional misconduct by another healthcare worker. In 85 % of these cases, the healthcare worker (suspected of) demonstrating incompetence or impairment was a nursing colleague. These findings do not imply that this proportion of nursing staff members actually *had* a nursing colleague who demonstrated impairment or incompetence as this partly refers to *suspicions* of professional misconduct. A survey study among ten healthcare professions regulated by law showed that 31 % of the healthcare professionals had experience with a colleague demonstrating impairment or incompetence in the preceding 12 months [3]. Our findings suggest that nursing staff in home care are slightly more likely to encounter a colleague who demonstrates actual or possible incompetence or impairment.

Earlier research among nursing staff has shown that nurses in hospitals and nursing homes are rather likely to observe *acts* of inadequate care or disruptive behaviour [6–8]. The current findings reveal that experience with actual or possible professional misconduct by colleagues, as a (generally) lasting situation, is quite common among nursing staff in the home-care sector. This is remarkable since home-care nursing staff deliver care rather independently and generally out of sight of colleagues. The autonomous and self-reliant character of the work and lack of direct interference and close supervision of managers [23] may increase the risk of professional misconduct. However, this could also increase the risk of unjustified suspicions of professional misconduct as colleagues may lack a good picture of the care provided by their fellow workers.

Since home-care staff apparently quite regularly suspect a nursing colleague of impairment or incompetence, it is even more important that they report their suspicions of professional misconduct. The current study indicates that 20 to 35 % of the nursing staff experience difficulty in reporting suspicions of different types of incompetence and 25 to 52 % experience difficulty in reporting suspicions of various forms of impairment. The hesitancy of some nursing staff members to report acts of inadequate care, as shown by Firth-Cozens, et al. [10] and Moore and McAuliffe [6], seems to apply to reporting suspicions of professional misconduct by nursing colleagues as well.

Our findings show that nursing staff experience more difficulty in reporting suspicions of misconduct associated with impairment than suspicions of misconduct associated with incompetence. This is in accordance with Moore and McAuliffe [24], who found that 79 % of the hospital nurses who observed an incident of incompetence reported it, while only 61 % of the nurses who observed poor treatment/abuse reported this. Our study revealed that suspicions of misconduct associated with impairment are rather rare in comparison with suspicions of misconduct associated with incompetence. In addition to impairment as a taboo subject and a problem that is less obvious than incompetence, unfamiliarity with nursing colleagues being impaired could explain why nursing staff experience more difficulty in voicing suspicions of misconduct related to impairment than in reporting suspicions of misconduct associated with incompetence.

As expected, the results reveal that nursing staff with managerial tasks experience less difficulty in reporting suspicions of professional misconduct by nursing colleagues, both in cases of incompetence and of impairment. This finding is in line with the findings of Moore and McAuliffe [6], who found that nurse managers are more likely to report incidents of poor care than staff nurses in hospitals.

Our study indicates that nursing staff with a higher level of education (registered nurses with a bachelor’s degree) experience less difficulty in reporting suspicions of incompetence than nursing staff with a lower level of education (registered nurses with an associate degree and certified nursing assistants). This is consistent with Malmedal, et al. [17] who revealed that nursing-home staff with a lower level of education feel less brave about reporting acts of inadequate care than more highly educated nursing staff. However, surprisingly, no relationship was found between educational level and difficulty in reporting suspicions of impairment. Possibly, as a professional group, registered nurses with a bachelor’s degree may be more accustomed to exerting social control over the professional behaviour of colleagues than to exerting social control over personal problems.

Contrary to expectations, we found no associations between, on the one hand, work experience and prior experience with professional misconduct and on the other hand difficulty in reporting professional misconduct. Malmedal, et al. [17] showed that nursing-home staff with more than 30 years of work experience are more afraid of the personal consequences of reporting *acts* of inadequate care committed by their colleagues than staff with less experience. This does not seem to apply to reporting suspected misconduct by nursing colleagues in home care.

It is of great importance that professional misconduct by nursing staff is avoided as far as possible. This study has found that three-quarters of the nursing staff consider a positive team climate important in preventing professional misconduct. Furthermore, two-thirds attach great value to discussing incidents and 57 % believe good communication between healthcare workers is critical. We found no association between on the one hand the importance nursing staff attach to these three aspects of professional practice and on the other hand educational level and prior experience with professional misconduct. Both nursing staff with higher education levels and those with lower education levels, and both nursing staff with actual experience with suspected misconduct and those without think that a positive team climate, discussing incidents and good communication between healthcare workers are important aspects of professional practice that help prevent professional misconduct.

### Implications

Home-care employers and professional associations should encourage nursing staff to report suspicions of impairment or incompetence concerning their nursing colleagues in order to tackle professional misconduct by nursing staff. This can be achieved by eliminating the barriers nursing staff may encounter when they attempt to raise an issue. These barriers vary between different types of nursing staff. Employers and professional associations should therefore tailor their approach to the educational level and managerial tasks of the nursing staff concerned. In their study of nurses’ and doctors’ attitudes to reporting poor care, Firth-Cozens, et al. [10] conclude that in order to foster a reporting culture, there is a need to “*introduce clarity into the areas which must be reported and clear systems for doing so, ensure that mechanisms exist to bring about necessary change following reporting, and assure safety for those who have the courage to report”* (p.336). To respond to this need, home-care employers can draw up a protocol for dealing with nursing colleagues demonstrating incompetence or impairment. Professional associations can foster this by developing a model protocol. In the Netherlands, the associations of general practitioners and medical specialists have drawn up such a model protocol [25]. However, to date, a model protocol for home-care nursing staff is lacking and it is unknown whether individual home-care organisations have a protocol for dealing with nursing colleagues demonstrating impairment or incompetence. An important part of the organisational arrangements for reporting suspicions of professional misconduct is blame-free reporting. Previous research indicates that fears about negative consequences are an important barrier to reporting healthcare incidents [6, 26]. In addition, it could be useful to enhance nursing staff’s training in critically reflecting upon practice and in knowledge about ethics and moral practice. The results of our study indicate that this is particularly relevant for certified nursing assistants and registered nurses with an associate degree.

Advocating a positive team climate within nursing teams, encouraging nursing staff to discuss incidents and facilitating this, and promoting good communication between healthcare workers may be appropriate strategies for employers, team leaders and professional organisations to help reduce professional misconduct by nursing staff. Home-care nursing staff consider these aspects of professional practice most important in preventing professional misconduct. In order to justify this approach, home-care organisations and professional associations can examine whether organisations that focus on these aspects of nursing staff’s professional practice do indeed experience less professional misconduct.

### Limitations and suggestions for future research

Although the first part of our study considered *actual* observations or suspicions of healthcare workers demonstrating incompetence or impairment, nursing staff were asked about *hypothetical* scenarios of suspected professional misconduct in the second part of the study. Jones and Kelly [27] consider the focus on hypothetical scenarios and intentions to be a drawback of many studies in relation to whistle-blowing by nurses. It remains unknown to what extent home-care nursing staff’s expectations of difficulty in reporting suspicions of professional misconduct correspond to the actual difficulty they experience in a situation of suspected impairment or incompetence. Another limitation of this study is that we did not explore nursing staff’s reasons underpinning the difficulty they experience in reporting professional misconduct. Furthermore, certified nursing assistants and younger nursing staff were underrepresented in our sample. However, we found no association between experience with actual or possible professional misconduct and educational level or age. Finally, we used a Dutch sample and home-care nursing staff’s experience with professional misconduct may differ across countries. Nonetheless, it is likely that the associations found between the type of misconduct (either associated with impairment or with incompetence) and individual characteristics of nursing staff on the one hand and difficulty in reporting suspicions of professional misconduct on the other hand are also true for other countries.

Further research is needed to better understand the factors that might deter nursing staff from reporting suspicions of professional misconduct, for instance prior experiences with reporting. It is recommended that future research addresses actual suspicions of professional misconduct by nursing colleagues rather than hypothetical scenarios. Furthermore, including patients’ experiences with professional misconduct in future research is important in order to obtain a better view of the prevalence of professional misconduct in home care.

## Conclusions

This study showed that observations or suspicions of professional misconduct by colleagues occur quite frequently among nursing staff. However, a substantial proportion of the nursing staff experience difficulty in reporting suspicions of professional misconduct, especially in the case of suspected impairment. Nursing staff consider a positive team climate, discussing incidents and good communication between healthcare workers to be important aspects of professional practice in preventing professional misconduct among nursing staff.

## Abbreviations

CNA: Certified nursing assistant
RN-a: Registered nurse, associate-level degree
RN-b: Registered nurse, bachelor’s degree
S.D.: Standard deviation
